# HARTNUP DISEASE

**DOI:** 10.4103/0019-5154.39740

**Published:** 2008

**Authors:** A B Patel, A S Prabhu

**Affiliations:** *From the Department of Pediatrics, Clinical Epidemiology Unit, Indira Gandhi Government Medical College, Nagpur, India*

**Keywords:** *Hartnup disease*, *pellagra*, *tryptophan*

## Abstract

A 10 year old girl presented with clinical signs and symptoms of the triad of niacin deficiency namely skin eruptions, ataxia, mental changes and diarrhea. Although this deficiency could be nutritional where maize is a staple diet, this patient had neutral aminoaciduria which indicated a defective transport of neutral amino acid transporter in the kidneys and intestine resulting in failure of transport of tryptophan and other neutral (ie, monoaminomonocarboxylic) alpha-amino acids in the small intestine and the renal tubules.

Hartnup disease is an autosomal recessive disorder caused by the defective transport of neutral (i.e., monoaminomonocarboxylic) amino acids in the small intestine and the kidneys. Patients present with pellagra-like skin eruptions, cerebellar ataxia and gross aminoaciduria. We present here a 10-year-old girl from a consanguineous marriage, who was studying well till the development of diarrhea off and on as per informants with partial relief due to antimotility agents over a month and, psychiatric symptoms, confusion, agitation, abnormal tone of speech, abnormal flinging of the limbs over 15 days. She was afebrile, and vitals were stable. There was a skin rash in the form of crusted, fissured hyperpigmented well defined plaques over the dorsum of hands and legs (Figs. 1 and 2) (pellagrous glove and boot) face - the upper eyelid and the back of the ear (Fig. 3) was spared. Desquamation over the borders of the soles on both the sides was observed and this was associated with an intense burning sensation. She had very brisk tendon jerks but babinski's reflex was absent. Tongue showed the atrophy of the papillae. She was confused, but could give relevant answers to simple questions. She was diagnosed as niacin deficient based on the presence of the typical dermatitis, diarrhea and dementia; however, her dietary history (maize as a staple diet) did not suggest nutritional pellagra. Therefore, we suspected Hartnup disease. Her urine showed neutral aminoaciduria and she had hypochromic microcytic anemia. The skin, psychiatric symptoms and diarrhea responded dramatically to niacin 50 mg twice a day and she could be discharged after 7 days.

**Fig. 1:**
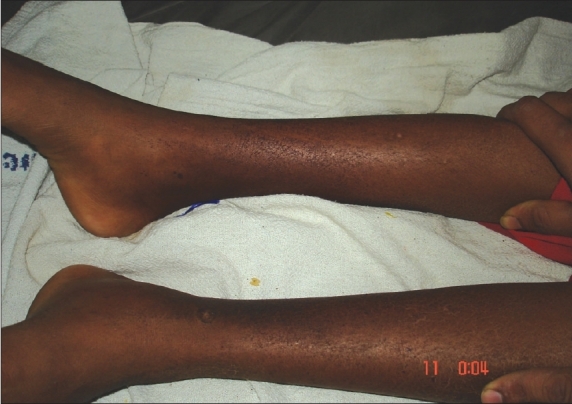
Lesions on lower limb

**Fig. 2:**
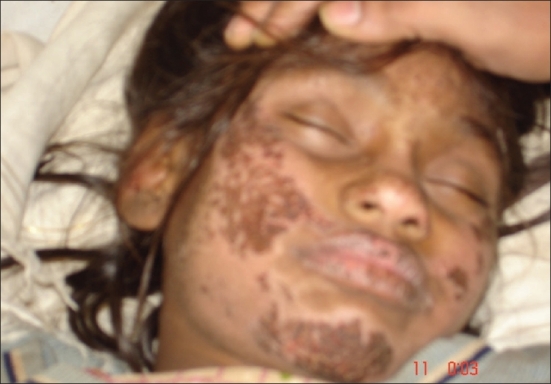
Sun-exposed lesions on the face, sparing the eyelids and upper surface of the orbit

**Fig. 3:**
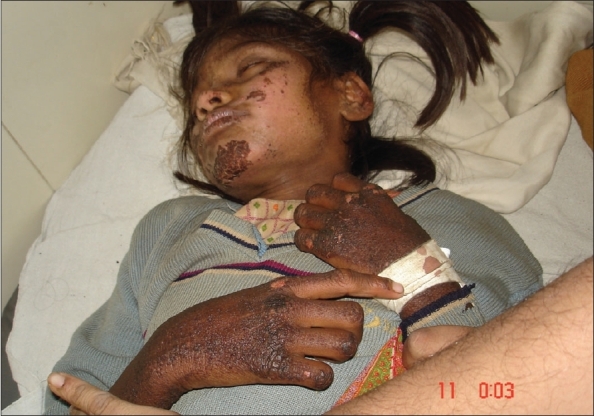
Lesions on the hands and face; hyperpigmented and crusted plaques with fissures

Hartnup was first described in four of the eight family members (Hartnup family of London) with aminoaciduria, a skin rash resembling pellagra and cerebellar ataxia by Baron *et al.*^1^ The causative gene, *SLC6A19,* is located on a locus on short arm of chromosome 5 (band 5p15.33) which encodes a transporter.^2^ The transporter is found in kidney and intestine, where it is involved in the resorption of all neutral amino acids. This sodium-dependent and chloride-independent neutral amino acid transporter is expressed predominately in the kidneys and the intestine resulting in the failure of the transport of tryptophan and other neutral alpha-amino acids (i.e., monoaminomonocarboxylic) in the small intestine and the renal tubules resulting in its presence in feces and aminoaciduria. An abnormality in tryptophan transport leads to niacin deficiency, which is responsible for the pellagra-like eruptions and photosensitivity. Amino acids (tryptophan) retained within the intestinal lumen are converted by bacteria to indolic compounds toxic to the nervous system. After absorption, indole is converted to 3-hydroxyindole (i.e., indoxyl, indican) in the liver where it is conjugated with potassium sulfate or glucuronic acid, then excreted by kidneys as indicanuria. Other tryptophan degradation products, including kynurenine and serotonin, are excreted in the urine as well. Other skin conditions that resemble this rash are seborrheic eczema (has yellow crusting), nutritional pellagra (where the staple diet is maize), congenital poikilodermas with photosensitivity, lupus erythematosus and carcinoid syndrome (associated with flushing).
